# After the Storm: Persistent Molecular Alterations Following HCV Cure

**DOI:** 10.3390/ijms25137073

**Published:** 2024-06-27

**Authors:** Coline Seurre, Armando Andres Roca Suarez, Barbara Testoni, Fabien Zoulim, Boyan Grigorov

**Affiliations:** 1 INSERM U1052, CNRS 5286, Centre Léon Bérard, Centre de Recherche en Cancérologie de Lyon, Université Claude Bernard Lyon 1, 69434 Lyon, France; coline.seurre@inserm.fr (C.S.); armando-andres.roca-suarez@inserm.fr (A.A.R.S.); barbara.testoni@inserm.fr (B.T.); fabien.zoulim@inserm.fr (F.Z.); 2 The Lyon Hepatology Institute EVEREST, 69003 Lyon, France; 3 Hospices Civils de Lyon, 69002 Lyon, France

**Keywords:** HCV, DAA, liver disease

## Abstract

The development of direct-acting antivirals (DAAs) against hepatitis C virus (HCV) has revolutionized the management of this pathology, as their use allows viral elimination in a large majority of patients. Nonetheless, HCV remains a major public health problem due to the multiple challenges associated with its diagnosis, treatment availability and development of a prophylactic vaccine. Moreover, HCV-cured patients still present an increased risk of developing hepatic complications such as hepatocellular carcinoma. In the present review, we aim to summarize the impact that HCV infection has on a wide variety of peripheral and intrahepatic cell populations, the alterations that remain following DAA treatment and the potential molecular mechanisms implicated in their long-term persistence. Finally, we consider how recent developments in single-cell multiomics could refine our understanding of this disease in each specific intrahepatic cell population and drive the field to explore new directions for the development of chemo-preventive strategies.

## 1. Introduction

The development of direct-acting antivirals (DAAs) against hepatitis C virus (HCV) is one of the most remarkable stories in translational research. Since the discovery of HCV in 1989 [1], the scientific community was able to develop the necessary research and diagnostic tools that led to the design of therapeutic molecules, allowing HCV elimination in more than 95% of cases nowadays [2]. The success of DAAs has shown that it is possible to surmount the challenges associated with chronic liver infections, which has led to a renewed interest in the development of curative therapies for hepatitis B virus (HBV) and hepatitis delta virus (HDV). Nonetheless, HCV remains a global health burden due to several obstacles associated with its diagnosis, treatment availability and the difficulties of developing a prophylactic vaccine against this pathogen [3]. Moreover, and despite the improvement in long-term clinical outcomes observed in HCV-cured patients, a significant risk of hepatocellular carcinoma (HCC) development remains [4,5]. Thus, the present review explores the effect that DAA treatment has on the liver microenvironment, the alterations that persist after HCV cure and the mechanisms that can potentially explain the increased risk of liver complications in these individuals.

## 2. The HCV Cycle

HCV is a small enveloped virus with a positive single-strand RNA genome, which belongs to the *Flaviviridae* family and is the only member of the *Hepacivirus* genus [6]. The preferential HCV tropism for hepatocytes stems from a wide variety of host factors present in this cell type. Indeed, HCV entry is a complex multi-step process involving the E1/E2 glycoprotein heterodimer and lipoprotein components present in the viral envelope, which interact with various cellular factors that include low-density lipoprotein receptor (LDLR), heparan sulfate proteoglycans (HSPGs) such as syndecan-1, scavenger receptor class B type 1 (SR-BI), CD81 and claudin 1 (CLDN1) [7,8,9,10]. Binding to CD81 leads to the activation of signaling pathways that favor virion internalization, such as epidermal growth factor receptor (EGFR), and Rho and Ras GTPases [11,12], events that are further amplified by the viral manipulation of host factors (e.g., netrin-1) [13]. This tightly orchestrated entry process is followed by clathrin-mediated endocytosis and release of the HCV genome into the cytoplasm. In its 5’- untranslated region (5’-UTR), the HCV genome contains an internal ribosome entry site (IRES), which is recognized by ribosomal subunits in the endoplasmic reticulum (ER) [14,15]. HCV RNA undergoes translation, resulting in a viral polyprotein of about 3000 amino acids. Cleavage of this polyprotein also takes place within the ER. Initially, cellular proteases cleave the core, E1/E2 and p7 proteins. Then, the viral protease NS2 cleaves between itself and NS3. This is followed by NS3 cleavage of NS4A from itself and NS4B. Finally, the NS3/NS4A protease complex cleaves at the NS4B/5A and NS5A/5B junctions, resulting in the formation of 10 mature viral proteins [16]. 

HCV replication takes place exclusively in the cytosol, within ER-derived double-membraned vesicles (DMVs), the formation of which is induced by the NS5A phospho-protein [17]. These “viral factories” concentrate viral components, thus shielding them from innate sensors. NS5A binds the RNA-dependent RNA polymerase (RdRP) NS5B, the essential catalytic enzyme for HCV replication. It drives the formation of a negative-sense RNA intermediate, which serves as a template for generating the positive-sense HCV RNA used either for translation and replication or as a genome for the newly formed virions. Although the precise mechanisms implicated in viral assembly are not completely elucidated, it is suggested that lipid droplets (LDs), which are in close proximity to DMVs, constitute an assembly platform for HCV particle formation. The particles are released from cells as HCV lipoviral particles (LVPs) [17]. The released LVPs are rich in triglycerides and contain apolipoproteins B (ApoB) and E (ApoE) [18]. In this context, we have recently reported that HCV infection enhances the expression of heparanase-1 (HPSE-1), an enzyme present in the extracellular matrix that cleaves heparan sulfate chains from the heparan sulfate proteoglycans, and favoring HCV egress through CD63 exosome formation [19]. Thus, to promote its replication and spread, HCV needs to modulate host cell metabolism, particularly by inducing energy production and biomolecule synthesis. This metabolic hijacking is well-documented at the molecular level, as described in the sections below, leading to a wide variety of intra- and extra-hepatic alterations in HCV-infected patients. 

## 3. Impact of Chronic HCV Infection in the Liver Microenvironment

Given that approximately 70% of HCV-infected individuals develop chronic hepatitis C (CHC), it is not surprising that the virus has adapted its cycle to hijack host cellular pathways (e.g., lipid metabolism, exosome biogenesis) and efficiently handle the pressure of immune responses. Indeed, as a non-integrated RNA virus, HCV must be constantly replicating and escaping innate sensors that can trigger the action of antiviral effectors [20]. This results in different alterations in the liver microenvironment that can, in the long-term, lead to the development of metabolic complications, cirrhosis and HCC [21]. 

### 3.1. Metabolic Alterations Associated with HCV Infection

As mentioned in the section above, the HCV cycle is closely linked to lipid metabolism, since HCV virions present many components typically found in low-density lipoproteins (LDLs) and very-low-density lipoproteins (VLDLs), such as ApoE [22]. This important association can also be appreciated by the use of VLDL assembly inhibitors, which result in decreased HCV production [23]. Moreover, it has been shown that newly translated non-structural viral proteins induce the accumulation of LDs on the ER membrane. LDs are small organelles containing a lipid ester core, primarily composed of triglycerides and cholesterol esters, surrounded by a phospholipid monolayer [24]. Besides their role in intracellular lipid storage, LDs facilitate metabolic regulation and communication between organelles. HCV proteins modify the association between the ER and LDs, forming a membranous web that includes cholesterol-rich DMVs associated with cytoplasmic LDs, which serve as complexes for HCV replication and virion assembly [25]. At the molecular level, it has been reported that HCV induces the activation of inflammatory pathways such as signal transducer and activator of transcription 3 (STAT3), which impairs peroxisomal function via the downregulation of peroxisome proliferator activated receptor alpha (PPAR-α), ultimately leading to the accumulation of long-chain fatty acids and thus offering a potential replicative advantage to the virus [26].

These observations are of particular clinical relevance, as 40–80% of HCV-infected patients show an accumulation of lipids in the liver, a condition known as steatosis [27]. In particular, HCV genotype 3 infection is associated more frequently with steatosis compared to other genotypes, and its severity is proportional to HCV replication levels [28]. Dysfunctions in lipid metabolism can also trigger insulin resistance (IR) and type II diabetes mellitus (T2DM) in CHC patients, increasing the risk of fibrosis progression [29]. IR is characterized by either an inadequate amount of insulin to maintain glucose levels or the inability of normal insulin concentrations to maintain glucose homeostasis, resulting in elevated blood glucose levels [30]. Several factors might explain this glycemic elevation induced directly or indirectly by HCV infection. Regarding direct mechanisms, the HCV core protein upregulates phosphorylation of insulin receptor substrate (IRS1), leading to its degradation and thereby inhibition of the downstream insulin signaling pathway [31]. HCV also strongly upregulates the secretion of tumor necrosis factor alpha (TNF-α), which can affect IRS1 phosphorylation [32]. Moreover, HCV has been reported to downregulate glucose transporter type 4 insulin-responsive (GLUT4) expression, a protein responsible for glucose entry into cells [33]. In contrast, the expression of cellular components implicated in glutamine metabolism (e.g., glutaminase, GLS) is increased during HCV infection and has been linked to liver progression [34]. As described in the sections below, the molecular mechanisms implicated in these metabolic alterations are often equally relevant to the modulation of antiviral response against HCV. As an example, HCV NS5A interacts with glucokinase via its D2 domain, thus promoting glycolysis [35]. This very same D2 domain of NS5A can also impair immune response and suppress interferon signaling [36].

### 3.2. HCV-Induced Impairment of Immune Response

Upon infection, HCV is initially detected by innate immunity sensors within hepatocytes, such as retinoic acid-inducible gene I protein (RIG-I) [37]. This leads to the activation of local antiviral responses via the induction of interferons (IFNs) [38]. However, HCV has been described to inhibit downstream IFN signaling and limit the expression of interferon-stimulated genes (ISGs) [39,40,41]. This persistent and inefficient antiviral response in the liver microenvironment contributes to HCV persistence and pathogenesis such as tissue damage and hepatic complications including HCC [42,43].

The alteration of antiviral responses in the liver is not limited to HCV-infected hepatocytes, as the function of multiple immune cell populations is impacted, including many of the cell types that initiate innate responses against HCV, such as granulocytes (i.e., neutrophils, eosinophils, basophils), monocytes/macrophages and dendritic cells (DCs). During HCV infection, liver-resident macrophages (i.e., Kupffer cells, KCs) present an increased expression of activation markers such as CD163 [44], which is accompanied by the production of a series of cytokines with antiviral activity that include interleukin (IL)-1β and IFN-γ [45,46]. At the same time, viral proteins such as HCV core are implicated in the induction of inhibitory factors (e.g., programmed cell death 1 ligand 1, PD-L1, CD274) that can affect their functions. At the molecular level, this increased PD-L1 expression in response to HCV core was shown to be Toll-like receptor (TLR)-dependent and to require PI3K signaling [47]. A similar situation is described for DCs, as they present an impaired maturation status and capacity to stimulate T cells [48,49].

Natural killer (NK) cells are an abundant intrahepatic lymphoid population, which is also implicated in antiviral responses against HCV [50]. During the acute phase of infection, NK cells present an activated phenotype characterized by increased IFN-γ production and capacity to kill infected cells [51,52]. However, during the chronic phase, the phenotype and function of NK cells are altered. Several studies reported a decrease in the expression levels of killer cell lectin like receptor K1 (KLRK1, NKG2D), which is an activating receptor present on cytotoxic cells [53,54]. Mechanistically, Sène et al. reported that the NS5A HCV protein interacted with TLR4 on monocytes and induced the secretion of TGF-β, which then downregulated NKG2D in NK cells, leading to a reduction in their cytotoxic action [54]. Furthermore, the number of circulating NK cells is significantly lower in HCV-infected patients compared to healthy individuals [55].

Mucosal-associated invariant T (MAIT) cells, a type of innate-like T cell population that is highly present in the hepatic microenvironment [56], are also impacted during HCV infection. Similar to NK cells, their number is markedly decreased in the circulation of HCV-infected patients. However, the MAIT cells that persist have an activated phenotype with upregulation of granzyme B (GZMB), HLA-DR, programmed cell death 1 (PDCD1, PD-1) and CD69 expression, as well as suppressed responsiveness to major histocompatibility complex, class I-related (MR1)-dependent antigen stimulation, which suggests an impairment of this immune population due to HCV infection [57]. 

T-cell exhaustion due to the continuous stimulation with HCV antigens is a hallmark of CHC [58]. Exhausted T-cells lose their effector functions (e.g., cytokine production) and proliferative capacities, which can be associated with increased levels of inhibitory receptors such as PD-1, cytotoxic T-lymphocyte associated protein 4 (CTLA4), and hepatitis A virus cellular receptor 2 (HAVCR2, TIM-3) [59]. In addition, HCV-associated liver tumors are characterized by a global downregulation of genes involved in innate and adaptive immune responses, with T-cell activation as one of the most altered pathways [60]. 

All of these metabolic and immune HCV-induced alterations can be linked to the establishment of persistent liver damage, ultimately leading to the development of hepatic fibrosis and HCC. Regarding the available therapeutic options against the virus, several drugs have been developed to directly target HCV proteins and manage this chronic infection, affecting millions of people worldwide. 

## 4. DAA Therapy for Chronic HCV Infection

**Figure 1:**
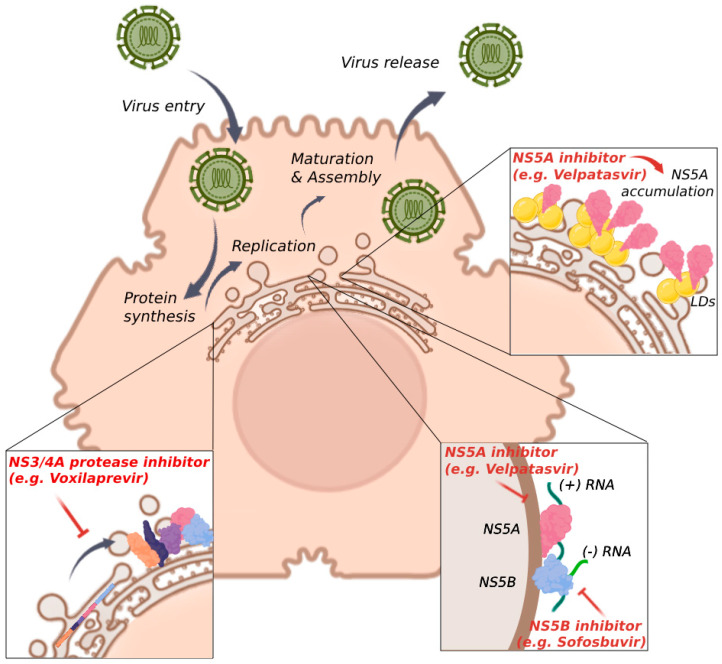
HCV cycle and the viral components targeted by DAA therapy. DAAs inhibit several HCV non-structural proteins, acting at different steps of the HCV cycle. NS3/4A inhibitors prevent polyprotein cleavage in the ER, thus blocking the formation of mature viral proteins. NS5B inhibitors target the RdRp, thereby inhibiting HCV translation and replication. NS5A inhibitors induce accumulation of this protein in the ER compartment, thus blocking its function during HCV replication and assembly. DAAs, direct-acting antivirals; ER, endoplasmic reticulum; HCV, hepatitis C virus; RdRp, RNA-dependent RNA polymerase; LDs, lipid droplets.

Management of HCV infection has fundamentally changed since 2011 with the introduction of DAAs. In this context, IFN-free DAA therapy leads to a sustained virological response (SVR) in 95% of patients, which is defined as undetectable HCV RNA in serum or plasma 12 weeks (SVR12) or 24 weeks (SVR24) after the end of therapy, as assessed by a sensitive molecular method [2]. SVR is frequently associated with improved liver functions for these patients. DAAs inhibit non-structural proteins, which are crucial for HCV replication. They are divided into three classes based on their specific target: NS3/4A protease (e.g., voxilaprevir), NS5A (e.g., velpatasvir) or NS5B (e.g., sofosbuvir) [61] (Figure 1). These drugs are taken as a combination therapy in order to increase the success rate of HCV elimination, with the choice of molecules depending on the medical history and health status of each patient. Moreover, many DAAs exhibit pan-genotypic efficacy, which facilitates treatment as it is not necessary to determine the HCV genotype and subtype [2]. 

## 5. Impact of DAA Therapy against HCV in the Liver Microenvironment

Despite the high SVR rates associated with DAA treatment, recent studies suggest that patients with HCV-associated liver damage, such as advanced fibrosis and cirrhosis, still face a considerably high risk of HCC development. Indeed, SVR leads to a reduction of de novo HCC incidence that is close to 80% [62,63]. Moreover, the yearly incidence of liver-related mortality in individuals with SVR is 0.36% (vs. 0.96% in non-SVR) [4,63]. The presence of metabolic risk factors such as obesity and T2DM are major determinants in the progression of CHC, even after HCV cure [64,65]. Thus, understanding the clinical and molecular alterations that persist following DAA treatment is of central importance. 

### 5.1. Modulation of Liver Metabolism Post HCV Cure

**Figure 2:**
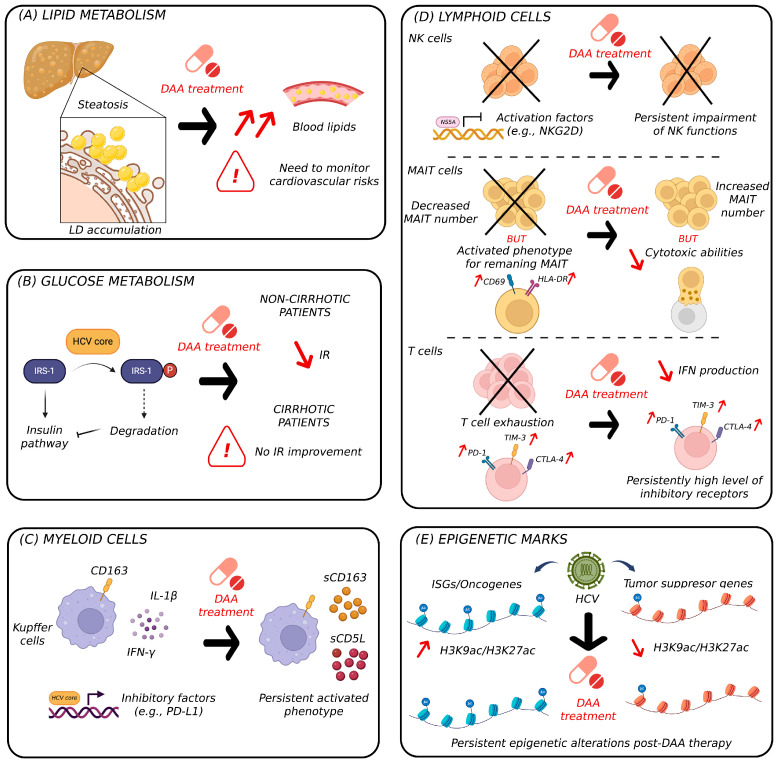
Persistent molecular alterations following HCV cure. (**A**) In HCV-cured individuals, blood lipid levels may return to normal or even increase after DAA treatment, potentially increasing the risk of cardiovascular events. (**B**) Depending on the patient’s fibrosis score, glucose metabolism improves in non-cirrhotic patients or remains altered in cirrhotic patients. (**C**) HCV infection impacts myeloid cells (e.g., Kupffer cells) and their phenotype is not fully reverted after DAA treatment, as high levels of activation markers can still be detected (e.g., sCD163). (**D**) HCV impairs the function of several lymphoid cell populations, which are not fully restored post-DAA treatment: the global NK receptor repertoire is not restored (top), the number of MAIT cells increases but their cytotoxic abilities remain altered (center) and T-cell exhaustion phenotypes persist (bottom). (**E**) The persistence of HCV-induced epigenetic alterations (e.g., H3K9ac, H3K27ac) on ISGs, oncogenes and TSGs could contribute to the development of immune and metabolic complications, despite HCV elimination. CTLA4, cytotoxic T-lymphocyte associated protein 4; DAAs, direct-acting antivirals; HCV, hepatitis C virus; IFN, interferon; IR, insulin resistance; IRS-1, insulin receptor substrate-1; ISGs, interferon-stimulated genes; LDs, lipid droplets; MAIT, mucosal-associated invariant T cells; NK, natural killer cells; PD-1, programmed cell death 1; PD-L1, programmed cell death ligand 1; TSGs, tumor suppressor genes.

It is well documented that HCV cure in IFN-treated patients is associated with an elevation in blood lipids [66,67]. Although studies on HCV-infected patients treated with DAAs are relatively recent, it has also been reported that a significant increase in plasma lipids can be observed several months after HCV eradication, including total cholesterol, LDL-C, VLDL and triglycerides [68,69,70,71]. Furthermore, achieving SVR has been significantly associated with weight gain in these patients [72]. The cause of this metabolic profile remains poorly characterized, but it has been suggested that it could stem from several factors that include an improved quality of life and increased nutritional intake [29,73]. Nonetheless, a lower incidence of cardiac events is associated with SVR achieved with DAAs, as compared to patients treated with pegylated IFN and ribavirin (RBV) [74] (Figure 2A). 

These retrospective studies also investigated glucose metabolism, given the crucial role of the liver in maintaining plasma glucose levels. There are conflicting data regarding the impact of DAAs on glucose homeostasis. IR can be assessed by measuring hemoglobin A1C (HbA1c) blood levels or by utilizing the homeostatic model assessment for insulin resistance (HOMA-IR) score. Morales et al. reported a significant decrease in HbA1c levels up to six months post-treatment with sofosbuvir-based regimens, particularly in diabetic patients [69]. These data were confirmed by Hum et al. who studied a cohort of 2435 diabetic patients undergoing IFN- and RBV-free DAA-based treatment, describing a significant reduction in HbA1c levels that was associated with improved glycemic control [75]. Lee et al. studied a cohort of 415 HCV-infected patients for three years and confirmed the above results using the HOMA-IR score, which decreased after 72 weeks of DAA therapy [76]. Noteworthy, Weidner et al. identified that for patients with liver cirrhosis, DAA treatment failed to improve glucose metabolism [77]. Thus, cirrhotic patients require continued monitoring in order to limit the impact of metabolic disorders (e.g., obesity, malnutrition), in spite of HCV eradication [2,78] (Figure 2B). 

### 5.2. Effect of HCV Cure on Intrahepatic Immune Responses

In a recent study, Cui et al. conducted a transcriptomic characterization at single-cell resolution of intrahepatic myeloid populations in patients before and after HCV eradication using DAAs [79]. This study highlights a series of cell-type specific modifications following HCV cure, with the most notable changes related to IFN signaling pathways. The authors established a correlation between a high viral load before DAA treatment and a low rate of ISGs post-treatment in all cell types. Regarding specific populations, an increased expression of PD-L1/L2 in neutrophils and indoleamine 2,3-dioxygenase 1 (IDO1) in eosinophils was observed in chronic HCV patients. The expression of these T cell-inhibitory factors decreased after DAA treatment. An additional main finding is the increase of CD1C^+^ proliferative DCs post-cure, which present increased MHC-II expression levels, suggesting the restoration of a competent immune system [80]. On the contrary, this study also described the upregulation of soluble CD163 (sCD163) and CD5 molecule like (CD5L), two serum markers for macrophage activation, suggesting the persistence of an inflammatory response even after HCV cure [79,80] (Figure 2C).

Regarding NK cells, there are conflicting reports describing the impact of HCV cure on this cell population. On one hand, some studies report that changes in NK cell homeostasis induced by chronic infection are restored after treatment with different DAA combinations [81,82]. On the other hand, Strunz et al. compared NK cell phenotypes pre- vs. post-DAA treatment in three independent cohorts, showing a global reduction in the NK cell receptor diversity compared to healthy controls [53]. Furthermore, Zhang et al. observed that the total amount of NK cells after DAA treatment remains unchanged, with fewer NK cells in HCV patients compared to healthy controls. This work also concluded that, while DAA therapy transiently improved the cytotoxic functions of NK cells in the first weeks of treatment, such an effect did not persist in the long term [83], suggesting a permanent impairment in immune homeostasis. In DAA-treated patients, the impairment of NK functions (e.g., antimicrobial immunity, cancer cell destruction) may be involved in HCC development (Figure 2D). 

Peripheral MAIT cells are considerably impacted by HCV infection, and their functions and number do not seem to be restored after DAA therapy [57,84]. Although DAA treatment is associated with an increase in the number of intrahepatic MAIT cells, this population shows a reduced cytotoxic capacity [85]. Considering that MAIT cells are important effectors in the response against pathogens [86], their persistent dysfunction after DAA treatment suggests an impaired immune surveillance in HCV-cured patients (Figure 2D). 

A single-cell transcriptomic characterization of peripheral T cell populations revealed a significant decrease in IFN signaling during and after DAA treatment, suggesting the restoration of circulating lymphocyte populations [87]. Nonetheless, it has also been reported that following DAA treatment, the cytokine production capacity of CD8^+^ T cells seems not to be restored, and there is no significant reduction in the expression of exhaustion markers such as PD-1 or TIM-3 [88] (Figure 2D). Similarly, Llorens-Revull et al. focused on HCV-specific T cells and revealed that CD4^+^ T cell exhaustion was not completely reversed, particularly in cirrhotic and older patients. The authors also observed a partial restoration of the proliferative capacities of these T cells after stimulation with NS3 helicase antigen, but not with the HCV core protein [89]. Taken together, these data demonstrate that, 3 months after DAA treatment cessation, the immune balance is not fully restored and the ability of the immune system to regain its functions is dependent on multiple factors, such as the severity of the disease and patient age. 

Overall, these studies characterizing the metabolic and immune status of patients after HCV cure using DAAs suggest that regular clinical monitoring is essential to prevent potential hepatic complications. 

## 6. Potential Molecular Mechanisms Implicated in the Persistent Risk of Liver Disease Post HCV Cure

Gene expression is tightly regulated by diverse epigenetic mechanisms that include modification of DNA (e.g., methylation) or its associated histones [90]. These latter modifications are commonly used as markers for chromatin accessibility and include acetylation of lysine 27 on histone 3 (H3K27ac) and lysine 9 on histone 3 (H3K9ac), as well as trimethylation of lysine 4 on histone 3 (H3K4me3), the three of them associated with euchromatin [91]. In this context, it has been shown that HCV infection induces persistent alterations in the host epigenome that persist after its eradication with DAAs [92,93,94]. In these studies, the analysis of histone modifications pre- versus post-DAA suggests a pattern that correlates with gene expression profiles known to be associated with and increased HCC risk [95]. 

In fact, H3K27ac deposition is increased in many genes associated with cancer hallmarks, such as the negative regulation of apoptosis (e.g., sphingosine kinase 1, SPHK1), or components implicated in inflammatory signaling, including TNF-α and IFN pathways [93]. These findings were confirmed and expanded in a subsequent study with CHC patients with metabolic dysfunction-associated steatohepatitis (MASH), describing the additional alteration of genes involved in cell cycle control (e.g., cyclin D2, CCND2), Notch signaling (e.g., mastermind like transcriptional coactivator 2, MAML2) and epithelial-mesenchymal transition (EMT) (e.g., cadherin 11, CDH11) [94]. Interestingly, both studies showed a decrease of H3K27ac deposition on tumor suppressor genes (TSGs), suggesting a downregulation of these preventive mechanisms implicated in the control of cancer development. Likewise, decrease in H3K27ac deposition was described in genes implicated in DNA damage responses (e.g., Fanconi anemia complementation group C, FANCC) or in the mTOR pathway (tuberous sclerosis complex 2, TSC2) [93,94]. Increased deposition of an active mark (i.e., H3K9ac) was identified in genes implicated in WNT/β-catenin (e.g., Wnt family member 10, WNT10), lipid metabolism (e.g., endothelin 1, EDN1), cytoskeleton remodeling and clathrin-coated vesicle formation [92] (Figure 2E).

The altered DNA methylation profile associated with HCV infection in vitro is not completely restored after elimination of the virus, as less than 15% of hypermethylation events were reversed by DAA treatment [96]. Among dysregulated genes, those linked to cell viability and apoptosis were the most frequently found. Consequently, supplementing DAA treatment with DNA demethylation agents to restrict the expression of potential oncogenes may reduce HCC risk [96]. 

These epigenetic alterations are not limited to HCV-infected hepatocytes, as the exhaustion of CD8*^+^* T cells has been associated with a specific epigenetic signature [97]. Moreover, several teams have reported that such a CD8*^+^* T cell “exhaustion signature” persists after DAA treatment, as chromatin accessible regions were retained long-term near classic exhaustion markers such as thymocyte selection associated high mobility group box (*TOX*) [97,98]. 

## 7. Conclusions

The landscape of therapeutic options against HCV has changed considerably in the past years with the development of DAA combination strategies, resulting in an improved quality of life and prognosis in these patients. Nonetheless, successful virus eradication seems not to completely restore the metabolic and immune balance in intrahepatic and peripheral cell populations [21,99], as HCV-cured individuals are still at risk of developing complications such as hepatic cirrhosis and HCC [63]. Additionally, this post-SVR risk is proportional to the existing liver damage and fibrosis score [62], supporting the surveillance of patients with advanced disease for early HCC detection [100]. Finally, it is worth mentioning the increased prevalence of metabolic dysfunction-associated steatotic liver disease (MASLD) in recent years [101], which could further alter the liver microenvironment in HCV-cured individuals and favor the development of complications.

As described in the sections above, multiple studies have highlighted how epigenetic modifications induced by HCV persist following DAA therapy [92,93,94,97,98]. Considering the recent developments in single-cell epigenomics [102], we now have the opportunity to refine our understanding of these alterations in each specific cell population of the intrahepatic microenvironment. This type of characterization has been performed recently in HBV-related HCC samples [103]. Therefore, similar studies would be highly relevant to explore specific questions such as the mechanisms behind HCC development in non-cirrhotic patients that have achieved SVR [104], as well as to identify potential prognosis markers and targets for novel chemo-preventive strategies in order to improve the management of HCV-cured individuals.
